# RETRACTION: Transmission Routes and a New Freshwater Crustacean Host for Infectious Precocity Virus (IPV)

**DOI:** 10.1155/tbed/9809502

**Published:** 2026-04-01

**Authors:** Transboundary and Emerging Diseases

RETRACTION: W. Xu, C. Zhao, X. Pan, X. He, X. Liu, J. Hou, and J. Pang “Transmission Routes and a New Freshwater Crustacean Host for Infectious Precocity Virus (IPV),” *Transboundary and Emerging Diseases*, 2023 (2023). https://doi.org/10.1155/2023/3950107.

The above article, published online on 4 December 2023 in Wiley Online Library (wileyonlinelibrary.com), has been retracted by agreement between the authors; the journal’s Editor‐in‐Chief, Dr. Zongfu Wu; and John Wiley & Sons Ltd.

The retraction has been agreed at the request of the authors due to the errors in the scientific content. Specifically, the authors identified that the data related to vertical transmission of IPV in Sections 3.1, 3.2, and 3.4 were inconsistent between the epidemiological and experimental host data. This directly affects the conclusions of the article, and it is therefore retracted.

The authors agree with this retraction.

